# *Helicobacter suis* Is Associated With Mortality in Parkinson's Disease

**DOI:** 10.3389/fmed.2019.00188

**Published:** 2019-08-28

**Authors:** Aisha D. Augustin, Antonella Savio, Amanda Nevel, Richard J. Ellis, Clive Weller, David Taylor, Rosalind M. Tucker, Mohammad A. A. Ibrahim, Ingvar Bjarnason, Sylvia M. Dobbs, R. John Dobbs, André Charlett

**Affiliations:** ^1^Pharmaceutical Sciences, King's College London, London, United Kingdom; ^2^The Maudsley Hospital, London, United Kingdom; ^3^Histopathology, Royal Marsden Hospital, London, United Kingdom; ^4^Royal Veterinary College, London, United Kingdom; ^5^Animal and Plant Health Agency, Weybridge, United Kingdom; ^6^Clinical Immunology, King's College Hospital, London, United Kingdom; ^7^Gastroenterology, King's College Hospital, London, United Kingdom; ^8^Statistics, Modelling and Economics, Centre for Infectious Disease Surveillance and Control, Public Health England, London, United Kingdom

**Keywords:** all-cause mortality, Parkinson's disease, *Helicobacter suis*, *Helicobacter pylori*, pig reservoir

## Abstract

*Helicobacter pylori* has been implicated in the pathogenesis of Parkinson's disease (PD). Its eradication, in a randomized placebo-controlled trial, improved PD hypokinesia. *Helicobacter* species zoonosis might explain excess mortality from PD and non-Hodgkin lymphoma in livestock, but not arable, farmers. Indeed, *Helicobacter* is causally-associated with gastric lymphoma. We have previously shown that the relative-frequency, *H. suis* to *H. pylori*, was 10-times greater in 60 PD-patients than in 256 controls. We now go on to evaluate the pathological significance of *H. suis*, detected in gastric-biopsy DNA-extracts by *ureA*-based species-specific qPCR, validated by amplicon sequencing. The methodology had been cross-validated by a *carR*-based PCR. The pathological significance is put in context of *H. pylori* detection [urea-breath-test (UBT) with biopsy-culture, and, if negative, PCR], and the potential reservoir in pigs. Here, we explore, in these 60 PD-patients, associations of *H. suis* status with all-cause-mortality, and with orthostatic cardiovascular and blood profiling. *H. suis* had been detected in 19 of the 60 PD-patients on one or more occasion, only two (with co-existent *H. pylori*) being UBT positive. We found that the hazard-of-death (age-at-diagnosis- and gender-adjusted) was 12 (95% CI 1,103) times greater (likelihood-ratio test, *P* = 0.005) with *H. suis*-positivity (6/19) than with negativity (2/40: one lost to follow-up). UBT-values did not influence the hazard. *H. suis*-positivity was associated with lower standing mean-arterial-pressure [6 (1, 11) mmHg], *H. pylori*-positivity having no effect. The lower total lymphocyte count with *H. pylori*-positivity [−8 (−1, −14) %] was not seen with *H. suis*, where T-cell counts were higher [24 (2, 52) %]. Regarding the potential zoonotic reservoir in the UK, *Helicobacter*-like-organism frequency was determined in freshly-slaughtered pigs, nature ascertained by sequencing. Organisms immunostaining for *Helicobacter*, with corkscrew morphology typical of non-*H. pylori Helicobacter*, were seen in 47% of 111 pig-antra. We conclude that *H. suis* is associated with all-cause-mortality in PD and has a potential zoonotic reservoir.

## Introduction

In the one randomized, double-blind, placebo-controlled trial (RCT) of the effect of biopsy-proven *Helicobacter pylori* eradication on the facets of Parkinson's disease (PD), we found sustained, clinically relevant improvement in objectively-measured hypokinesia (1, 2), providing 1b level causative evidence (3). Indication specificity of the improvement following *H. pylori* eradication is inferred from a surveillance study of antimicrobial exposure for any indication in PD (4). There is a lack of birth cohort effect for *H. pylori* anti-urease-antibody in PD (5), as in gastric cancer and peptic ulcer where causal links with *H. pylori* are generally accepted. Danish population registers show increased prescription of anti-Helicobacter drugs in the 5 years prior to PD diagnosis (6).

A single case of eradicating a corkscrew-shaped non-*Helicobacter pylori Helicobacter* (NHPH) has been described in a breath-test-positive PD-patient with antral gastritis (7). Biopsy-proven eradication in this cachectic patient, who had been wheelchair-bound for over a year, resulted in a U-turn in brady/hypokinesia, mastering previously neglected computing skills, and normalization of body mass index. Clinical relapse with return of breath-test positivity had occurred 14 weeks after giving a first-line anti-*H. pylori* regimen, but the lasting improvement followed exhibition of quadruple therapy containing oxytetracycline and bismuth. Indeed, subsequent susceptibility testing of *H. suis* isolates from sows suggests relative intrinsic insensitivity to amoxicillin and metronidazole, greater intrinsic susceptibility to tetracycline (8). Repeat biopsy in the PD-patient, 11 months after the quadruple therapy showed almost complete resolution of gastritis, with no organisms (7).

Subsequently, we found the relative-frequency of *H. suis* to *H. pylori*, to be 10-times greater in PD, when comparing gastric biopsies from 60 PD-patients with those from 256 routine gastroenterology patients (9) (*H. suis* frequency was standardized against that of *H. pylori* to avoid it being construed as true prevalence.) Results of the species-specific *ureAB* gene-cluster based RT-PCR employed were confirmed by sequencing the amplicon. The PCR detection method was cross-validated against an independent *carR*-based species-specific RT-PCR (10). Gastric-biopsy histopathology had not detected corkscrew *Helicobacter* in any of the 60 PD-patients (9). Improvement in hypokinesia following eradication of *H. pylori* in such “low density” (2, 11) indicates that detection of *Helicobacter* at PCR-only level is clinically important.

Zoonosis might explain the increased proportional mortality from PD among livestock farmers (but not in arable farmers) compared with all decedents, reported in a study of 26 USA states (12). Systematic review and meta-analysis of agrochemical usage have not resolved this difference between livestock and arable farming (13–15). An infective explanation is credible in a disease where neuroinflammation is an early feature (16) and peptic ulcer prodromal (11): NHPH is a candidate. However, gastric cancer mortality (17) was not increased in livestock farmers (12). Here, we go back to audit mortality and available clinical observations, including blood profile and cardiovascular measures, subsequent to NHPH detection in the 60 PD-patients (9), with a view to hypothesis-generation and future hypothesis-testing.

There may be a substantial reservoir for NHPH zoonosis: historically prevalence estimates in pigs at slaughter age in Europe, North and South America and Asia are around 60% (18–24). We assess the frequency of corkscrew *Helicobacter*-like organisms in a small sample (111) of UK pigs, using immunohistochemistry, backed-up by 16S rRNA gene amplicon sequencing (10 pigs) and shotgun metagenome sequencing (1 pig). In patients with gastric disease, the prevalence of NHPH, based on histopathology of gastric biopsy, has been estimated at between 0.2 and 6%, depending on geographical distribution. Low infection load, compared to *H. pylori* (25), militates against detection by [^13^C]urea-breath-test (UBT) and even histology.

Evidence points to facets of PD having different, not necessarily co-incident, drivers (26). Eliminating one aetiopathogenic marker might allow another to come to the fore. In the PD-patients audited here, frequency of *H. suis* on molecular microbiology was significantly greater where *H. pylori* had been eradicated (9). Similarly, the presence of *H. pylori* and small-intestinal-bacterial-overgrowth (SIBO) are inversely related in PD (27). We, thus, consider pathological associations of *H. suis* in the context of *H. pylori* and SIBO.

## Methods

### Audit Plan

Table 1 details the audit plan. It accesses routinely available data from patients with “clinically-definite” idiopathic parkinsonism (PD) (31), diagnosed at a clinic incorporating specialist gastrointestinal expertise. Sixty PD-patients were eligible on the basis that archived microbial DNA-extracts from their gastric biopsies had been included in a service re-evaluation of NHPH detection (with Gastrointestinal Reference Unit, Public Health England) (9). *Helicobacter suis* had been targeted since it was the most frequently reported NHPH in humans and a species-specific assay (*ureA*-based species-specific qPCR, validated by amplicon sequencing) was available (9).

**Table 1:** Audit topics and information required.

**Topic**	**Information required**
Demographic	Age, gender, height, weight, time-since-diagnosis, anti-parkinsonian medication.
Survival	Dates of any deaths and cause shown on death certificate, ascertained from relatives.
Cognitive function	Mini-mental state examination scores (28), with cut-point of <27/30 for cognitive impairment^*^.
Depression	Beck's Hopelessness scale score (29), with cut-points for mild (4–8/20), moderate (9–14) and severe (>14) depression.
Blood profiling	Full blood counts, leucocyte subset counts, serum haematinics (ferritin, folate, B12), homocysteine, and gastric autoantibodies.
Orthostatic cardiovascular monitoring	Pulse rates and blood pressures: lying, standing (immediate, 1 and 3 min) and post-exercise.
Lactulose hydrogen breath test	Status according to whether meter manufacturer's diagnostic cut–point (20 ppm increment) (30) exceeded in 2 consecutive readings^†^.
Nature/extent lifetime exposure to pigs and raw porcine products	History taken when *H. suis*-positivity fed-back following service re-evaluation^††^.

* *None had cognitive impairment at time of biopsy*.

† *Test followed 24 h deprivation of dairy-products/medicinal lactulose, and breakfast of a mug of clear fluid. Breath hydrogen measured (Gastrolyser, Micro Medical Ltd., Rochester, UK) before and after (15 min intervals for 2 h) 25G lactulose. Used in clinic as SIBO screen: if positive, fluid/fiber intake and use of maintenance bulk/osmotic laxatives encouraged. (Using glucose substrate would risk missing distal SIBO, since it is absorbed proximally)*.

†† *Option of re-biopsy offered at that time to those reported as positive*.

### Patients Audited

These were all of the PD-patients attending our National outpatient clinic, who had undergone gastroduodenal endoscopy over the decade before the service evaluation (9). The Gastrointestinal Reference Unit held archived microbial DNA-extract from all. Table 2 gives status for *H. pylori* and *H. suis*, based on first archived DNA-extracts. *H. suis* was present in 16 of the 60 PD-patients, *H. pylori* in 17, both species in 3. *Twenty* PD-patients had follow-up microbial DNA extracts from gastric biopsy in the archive. *H. pylori* was not detected at a follow-up biopsy in any of the 17 initially positive patients. However, 8/20 follow-up DNA-extracts were *H. suis-*positive, 5 remaining positive (4 despite an *H. pylori* eradication regimen) and 3 becoming positive *de novo* (after such a regimen). Thus, *H. suis* was detected in 19/60 patients on at least one occasion.

**Table 2:** Frequency of *H. suis* and *H. pylori* in first archived microbial DNA-extract from gastric biopsy in 60 PD-patients with and without known exposure to anti-*H. pylori* therapy [based on Bleacher et al. (9)].

**Frequency**	**Eradication category**	**Percent PD-patients with *Helicobacter* species (no. with species/total no. in category)**
*H. suis*	Previous exposure to anti-*H. pylori* therapy	58 (11/19)
	No known exposure	12 (5^*^/41)
	Total	27 (16/60)
H. pylori^**^	Previous exposure to anti-*H. pylori* therapy	0 (0/19)
	No known exposure	41 (17/41)
	Total	28 (17/60)

* *3 DNA-extracts also positive for H. pylori*.

** *H. pylori status for each period defined by UBT-, culture- or molecular microbiology-positivity. Where culture-negative, biopsies tested using PCRs targeting 16S rRNA (primer pair HP1/HP2) and vacA (Vac3624F/Vac3853R) genes. UBT not positive, without co-existing H. pylori, in any H. suis-positive patient*.

### Histopathology and Immunohistochemistry

Histological assessment was performed on 10% formalin-fixed, paraffin-embedded samples. Samples were oriented on cellulose acetate strips. Sections, 4 μm-thick, were cut perpendicular to mucosal surface, and stained with haematoxylin-eosin and immunostain with antibody generated against whole *H. pylori* lysate (VENTANA anti-*Helicobacter pylori* SP48 Rabbit Monoclonal Primary Antibody, Roche), using an automated slide-stainer (BenchMark XT, Roche). Slides were examined by light microscope at magnification × 4 to × 100. Preliminary testing showed that both the comma-shaped *H. pylori*-like and corkscrew organisms took up the immunostain.

Since human infection with NHPH is described as characteristically sparse and patchy (25), previous histopathology was subjected to service re-evaluation. Further sections were taken from archived wax-embedded initial or follow-up gastric biopsies from (i) the 6 patients with the highest density of *H. suis* DNA found (2 of whom were *H. pylori* culture-positive); (ii) 2 *H. pylori* culture-positive, but *H. suis* PCR-negative, patients, and (iii) 2 without evidence of *Helicobacter* (on UBT, histopathology, culture, or PCR).

One hundred and one pig stomachs (from 8 different UK farms) were sourced from an abattoir, immediately after slaughter, over a 2 month period, when both the team and pig stomachs available. On the first day, 10 stomachs were placed individually in sealed plastic bags. On the 2 subsequent days, stomachs were grouped by sealed bag according to farm. Ten old sow stomachs were sourced from the same abattoir on a separate occasion. Stomachs were opened, one at a time, along the major curvature. Contents were carefully removed manually, to retain as much mucus as possible. For the first 10 stomachs, a strip of mucosa, ⅓ cm wide, was dissected off the underlying muscle in each pars oesophogeal, cardia, fundus and antrum, and mounted in sequence on a cellulose acetate strip (Sartorius AG, Goettingen, Germany) using a sterile needle. Immunostaining results were used to determine the optimal sampling site for the subsequent 100 stomachs. Loaded acetate strips were all fixed in formalin within 4 h of slaughter. Any enlarged lymph nodes, lying along the lesser curvature of the first 10 stomachs, were dissected out and fixed.

### Molecular Microbiology

Caecal-content samples were collected in sterile containers from the first 10 pigs, to ascertain *Helicobacter* status, and, where gastric load of corkscrew organisms high, *H. suis* status (see Appendix) (32, 33).

### Statistical Analysis

Cox proportional-hazards models were used to assess which factors were associated with all-cause mortality. As with any mortality study, age and gender are *a priori* candidates for cofounding, and thus were included with no regard as to their statistical significance. Due to the limited number of events (deaths), apart from *H. pylori* infection and SIBO, which were deemed relevant to the aetiopathogenesis, no other covariates were considered because of the likelihood of over fitting. The proportional-hazard assumption was tested using an hypothesis-testing approach (34), as implemented in Stata 13s estat, phtest command. For all models fitted, the global test of the proportional-hazard assumption was not significant indication that this assumption was reasonable. Time of entry into the study was the date of presentation to clinic, when date of diagnosis of PD was noted if previous. Time of exit was date of death, or, if still alive, date of analysis. *H. suis* “status” (as evaluated September 2013) was considered as a fixed covariate regardless of date: a positive result being defined as molecular microbiology-positivity in any gastric biopsy from an individual. *H. suis* was considered as a fixed covariate regardless of date, because, at the time of this audit, non-invasive screening methodology to aid definition of presence/absence was not available. In contrast, *H. pylori* and SIBO status were each considered as time-varying covariates (35), constant within a specified period: (i) prior to any *H. pylori* eradication course; (ii) after that course and until any subsequent course; (iii) after any second course. Whilst all three are chronic, there are clear guidelines for establishing *H. pylori* and SIBO status (Tables 1, 2). Adjustment was made for the potential confounding variables, age at the beginning of each period, and gender.

Independent associations of the two *Helicobacter* species and SIBO with blood profiling and cardiovascular measures were assessed in mixed effects linear regression models, with blood or cardiovascular measures as continuous outcome variables, and adjustment for age and gender. Where necessary, log transformation was used to obtain an approximately symmetric distribution. A random intercept for each patient was included, to account for serial clinical measurements. *H. suis* status and, for each period, *H. pylori* and SIBO status were fitted as fixed effects.

## Results

Table 3 defines the PD-patients in whom association of *H. suis* with mortality and morbidity is studied. At the time of analysis, it was not determined whether one (*H. suis*-negative) of the original 60 patients was alive.

**Table 3:** Characteristics at start of audit period or earliest recorded after.

**Characteristic**		Mean (95% data interval)^*^ (*n* = 59)^**^
Demographic	Age (years)	63 (47, 80)
	Gender (male/female)	33/26^a^
	Height (m)	1.69 (1.52, 1.86)
	Weight (kg)	77 (51, 102)
	Time-since-diagnosis (years)	4 (0, 16)^b^
	Anti-parkinsonian medication^***^ (no/yes)	18/41^a^
	Medication includes levodopa (no/yes)	27/14^a^
Blood cell counts	Hemoglobin (g/L)	138 (116, 160)
	Red cell count (10^12^/L)	4.6 (3.8, 5.3)
	Total white cell count (10^9^/L)	6.3 (3.8, 12.0)^b^
	Neutrophils (10^9^/L)	3.9 (1.5, 8.0)^b^
	Lymphocytes (10^9^/L)	1.7 (0.7, 2.8)
	Platelet count (10^9^/L)	236 (154, 436)^b^
	Mean platelet volume (fL)	10.2 (7.4, 13.0)
Haematinics & homocysteine	Serum ferritin (μg/L) Serum folate (μg/L)	93 (7.4, 374)^b^ 408 (131, 985)^b^
	Serum B12 (ng/L)	443 (104, 1030)
	Serum homocysteine (μmol/L)	14.4 (7.4, 32.5)^b^
Gastric autoantibodies	Anti-intrinsic factor (–ve/+ve)	41/17^a^
	Anti-parietal cell (–ve/+ve)	45/13^a^
Mean arterial pressure	Lying (mmHg)	94.4 (65.9, 112.8)
	Immediate standing	95.3 (67.8, 122.8)
	1 min	96.9 (68.3, 125.4)
	3 min	96.6 (68.5, 125.3)
	Post-exercise	101.6 (69.5, 133.6)
Pulse	Lying	67.2 (48.3, 86.0)
	Immediate standing(/min)	76.8 (56.4, 97.1)
	1 min	77.4 (55.8, 99.0)
	3 min	75.9 (53.9, 97.8)
	Post-exercise	87.7 (64.2, 111.3)
SIBO	Hydrogen breath-test status (–ve/+ve)^****^	22/29^a^

* Except where denoted by superscript letter:

a Counts;

b *Centiles: 50 (2.5, 97.5)th*.

** *n = 48 for autoantibodies, 51 for SIBO*.

*** *Anti-parkinsonian medication used: amantadine, cabergoline, levodopa-combination, pramipexole, selegiline, rasagiline, trihexyphenidyl (low dose). Levodopa was combined with extracerebral dopa-decarboxylase inhibitor ± catechol-O-methyl transferase inhibitor, as evenly spaced as practicable to avoid iatrogenic fluctuations in performance. None were receiving a levodopa combination as monotherapy*.

**** *Forty two patients hydrogen-breath-test positive at some point during audit period*.

### Mortality Associated With *H. suis*

Age of decedents at diagnosis of PD had a mean of 59 (data interval 43, 76) years, that of survivors being similar [58 (41, 76) years]. The proportions of males were also similar: 3/8 and 30/51, respectively. The *H. suis*-positive had a mean age at PD diagnosis of 57 (38, 76) years, the *H. suis*-negative 58 (41, 75) years, corresponding proportions of males being 13/19 and 20/40.

Crude all-cause mortality was greater in those with *H. suis* (log-rank test, *p* = 0.006), with 6/19 of those positive at any time during follow-up having died, compared with only 2/40 of the rest. No deaths were attributed to gastric malignancy or peptic ulceration. Causes were other malignancy (3), infection (4, with dementia in 1), and dementia (1). Table 4 shows that the estimated hazard ratio of death during follow-up for the *H. suis*-positive was 12.1 [95% confidence interval (CI) 1.4, 103.1] (Cox proportional-hazards model, adjusted for age-at-diagnosis and gender: likelihood ratio test, *p* ≤ 0.005, neither age or gender reaching statistical significance at 0.05 level). UBT-values did not influence hazard.

**Table 4:** Estimated hazard ratios for Cox proportional-hazards model.

**Characteristic**	**Hazard ratio (95% C.I.)**	***p-value***
*H. suis* (positivity)	12.1 (1.4, 103.1)	0.02
Gender (female)	0.3 (0.1, 1.3)	0.1
Age at diagnosis (per year)	1.04 (0.95, 1.15)	0.4

**Figure 1:**
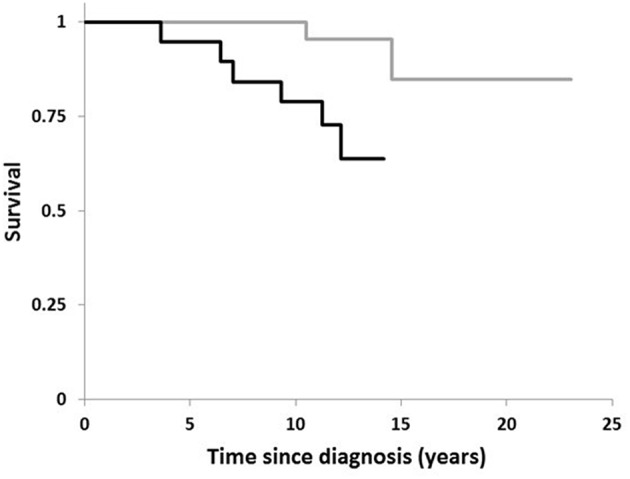
Comparison of survival in PD by *Helicobacter suis* status. Kaplan-Meier plot for 19 PD-patients with *H. suis* detected in DNA extract from gastric biopsy (black line) compared with that for the 40 in whom biopsy was negative for *H. suis* (gray line).

Figure 1 plots the Kaplan–Meier estimate of the survival function for the two conditions, *H. suis*-positivity and -negativity. The relatively short time-scale over which the deaths in the *H. suis*-positive occurred is striking. The first death in the *H. suis*-positive group was 3½ years after diagnosis of PD, the subsequent 5 losses occurring by 12 years, that is, on average, at 21 monthly intervals. In contrast, the first death in the *H. suis*-negative was not until 10½ years after diagnosis, the second at 14½ years.

*H. pylori-*positivity was not associated with mortality. Any association with SIBO-positivity could not be assessed since 5/8 deaths had occurred before hydrogen breath testing was used routinely in clinic.

### Cognition and Affect in Relation to *H. suis* Status

Fourteen of the 59 patients traced developed cognitive impairment (fall in mini-mental score to <27/30) whilst attending clinic or were diagnosed as having dementia after last seen there. The observed number of deaths in those with and without incident cognitive impairment (2 and 6, respectively) was not significantly different from number expected were death and cognitive impairment unrelated (log-rank test, *p* = 0.8). Cognitive impairment occurred in 8/19 *H. suis*-positive patients and 6/40 *H. suis*-negative: odds for being *H. suis*-positive tended to be higher [2.8 (95% CI 0.8, 9.5), *p* = 0.1] in those who developed cognitive impairment.

The majority of the PD-patients (82%) were classified as having at least mild depression (≥4/20) at some time during follow-up. There was no evidence of association between mortality and depression score [estimated hazard ratio 0.95 (0.83, 1.10), adjusted for age, gender and *H. suis, p* = 0.5]. The odds of being *H. suis*-positive was weakly (*p* = 0.1) related to the worst depression score recorded per individual, 4/5 patients with severe depression (score >14/20) being *H. suis*-positive (*p* = 0.05).

### Differential Effect of *H. suis* Status on Blood Profile

Table 5A illustrates the differential effect of *H. suis* on blood indices. *H. suis*-positivity was associated with a markedly higher (by 24%) T-cell count, through an effect on both CD4+ and CD8+ subsets. This contrasts with a lower lymphocyte count with *H. pylori* (8%), and a lower neutrophil count (23%) but numerically much higher natural killer cell count (40%) with SIBO.

**Table 5:** Independent associations of two *Helicobacter* species and SIBO with **(A)** total white cell and subset counts and **(B)** cardiovascular measures.

**A**						
**Cell count**	***H. pylori*****-positivity (*****n*** **=** **16/60)**		***H. suis*****-positivity (*****n*** **=** **17/60)**		**LHBT-positivity (*****n*** **=** **29/51)**	
	**Size effect**	***p*****-value**	**Size effect**	***p*****-value**	**Size effect**	***p*****-value**
	**Mean (95% CI)%**		**Mean (95% CI)%**		**Mean (95% CI)%**	
White cell count	2 (−4, 9)	0.5	6 (−7, 22)	0.4	−16 (0, −30)	0.05
Neutrophil	6 (−4, 16)	0.3	4 (−14, 25)	0.7	−23 (−1, −40)	0.04
Lymphocyte	−8 (−1, −14)	0.02	12 (−4, 32)	0.1	−8 (−26, 13)	0.4
T-cell (CD3+)	0 (−10, 10)	0.9	24 (2, 52)	0.04	−8 (−30, 22)	0.6
CD4+ T-cell	−3 (−12, 8)	0.6	20 (−1, 46)	0.06	9 (−16, 43)	0.5
CD8+ T-cell	−1 (−11, 11)	0.9	38 (2, 87)	0.04	−26 (−51, 11)	0.2
B-cell (CD19+)	−3 (−19, 16)	0.7	21 (−10, 61)	0.2	6 (−30, 59)	0.8
Natural-killer (CD16+CD56+)	−4 (−17, 12)	0.6	−12 (−33, 16)	0.4	40 (−4, 106)	0.08
**B**						
**Measurement**	***H. pylori*****-positivity**		***H. suis*****-positivity**		**LHBT positivity**	
	**Size effect**	***p*****-value**	**Size effect**	***p*****-value**	**Size effect**	***p*****-value**
	**Mean (95% CI)**		**Mean (95% CI)**		**Mean (95% CI)**	
Mean arterial pressure (mmHg)						
Lying	0 (−2, 2)	0.9	−4 (−9, 1)	0.08	3 (−5, 10)	0.5
Standing immediate	−1 (−3, 1)	0.5	−6 (−11, −1)	0.03	4 (−4, 11)	0.4
1 min	0 (−2, 2)	0.8	−6 (−11, −1)	0.03	2 (−6, 10)	0.7
3 min	0 (−2, 2)	0.8	−6(−11, −1)	0.02	2 (−6, 10)	0.6
Post-exercise	1 (−3, 2)	0.7	−6 (−12, 0)	0.07	4 (−5, 14)	0.4
Pulse (/min)						
Lying	0 (−1, 2)	0.8	1 (−4, 5)	0.8	−7 (−13, −1)	0.03
Standing immediate	0 (−2, 2)	0.9	0 (−5, 5)	0.99	−9 (−16, −3)	0.003
1 min	1 (−2, 3)	0.5	0 (−5, 5)	0.9	−9 (−16, −2)	0.008
3 min	1 (−1, 3)	0.5	0 (−5, 5)	0.9	−7 (−14, −1)	0.03
Post-exercise	0 (−3, 2)	0.8	0 (−7, 6)	0.9	0 (−9, 8)	0.9

Mean hemoglobin concentration and red cell and platelet counts were not associated with *Helicobacter* species or SIBO status. Mean platelet volume (10.2 fL) was toward the upper end of the reference range, larger with *H. pylori*-positivity [by 0.56 (0.07, 1.04) fL, *p* = 0.02]. Serum folate was lower [32 (15, 45)%, *p* = 0.001] with *H. pylori*-positivity. No other association with haematinics or with serum homocysteine was identified. *Helicobacter-*positivity, in general, was associated with anti-intrinsic factor gastric autoantibody, both *H. pylori*- and *H. suis*-positivity contributing to the effect [odds ratio 11 (1, 144) & 8 (1, 91), *p* = 0.06 & 0.09, respectively], only *H. suis*-positivity tending to be associated with parietal cell antibody [odds ratio 5 (1, 36), *p* = 0.1].

### Differential Effect of *H. suis* Status on Cardiovascular Measures

Table 5B illustrates differential effect of *H. suis* on mean arterial pressure and pulse rate. With *H. suis*-positivity, only, mean arterial pressure was lower, and this by a clinically-relevant amount (6 mmHg), for all standing measurement times. Lying and post-exercise values were numerically lower by a similar amount. With SIBO, only, lying and standing pulse rates were lower (8 beats/min), an effect lost on exercise.

### Previous Exposure to Pigs and Porcine Products in the *H. suis* Positive

Information on exposure to potential sources of *H. suis* was available in 11 of the 19 *H. suis-*positive patients. Of the five with exposure, two had daily contact with pigs (whilst growing up on farms), two had occasional contact (maximum once every month, during childhood), and one had worked in a butcher's shop as a youth. Otherwise, there was no recollection of contact with pig(s), handling raw pig products (other than in a domestic setting) or eating raw pig products.

### Histopathology and Immunohistochemistry in Patients

Presence of *Helicobacter* was confirmed in immuno-stained sections from *H. pylori* positive “control” patients, absence confirmed in negative controls. No *Helicobacter* staining was seen in sections from 4 of the 6 patients with the highest abundance of *H. suis* DNA. However, in the 2 others, who had evidence of both *Helicobacter* species, irregularly-shaped immuno-stained *Helicobacter*-like organisms (HLOs) were attached to the mucosal surface. In one of these, most HLOs were thicker than usual for *H. pylori* and tended to bank-up in irregular bundles, the rest being of typical *H. pylori* morphology. In the other, the HLOs also tended to bank-up, but were smaller, more homogenous.

### Histopathology, Immunohistochemistry and Molecular Microbiology in Pigs

Table 6A shows detection and density of immuno-stained corkscrew organisms, by gastric region sampled, in the 10 initial pig stomachs. Antrum was evidently the optimal sampling site. Enlarged lymph-nodes, found along the lesser curvature of stomach in 5 pigs, were immuno-stain-negative, despite 4 of them having obvious gastric *Helicobacter*. Table 6B compares frequency of detection and density of HLOs in antra from the 91 subsequent pigs (number 11–101) and from 10 adult sows (102–111). Immunostained corkscrew organisms were seen in 47% of pig antra. There was no difference in detection frequency between pigs and adult sows.

**Table 6:** Presence and density of *Helicobacter* on immunostaining in 111 UK pigs at slaughter, **(A)** by gastric region and in any enlarged lymph-nodes in initial pigs (1–10), and **(B)** in antra of pigs (1–101), and adult sows (102–111).

**A**						
**Pig no**.	**Anatomical site**					
	**Para-oeshageal**	**Cardia**	**Fundus**	**Antrum**	**Lymph-node**	
1	0	0	0	0	n/a	
2	0	++	^a^	+++	n/a	
3	0	+	0	++	0	
4	0	+	^a^	++	0	
5	0	^a^	^a^	+++	n/a	
6	0	^a^	0	^a^	n/a	
7	0	^a^	0	+++	n/a	
8	0	0	0	0	0	
9	0	+	++	^a^	0	
10	0	+	+	+++	0	
**B**						
**Pig no**.	**No. pigs with** ***Helicobacter*****-like organism in each density category**					**Total no**.
	**0**	^**a**^	**+**	**++**	**+++**	
1–10	2	2	0	2	4	10
11–101	38	12	15	14	12	91
102–111	3	2	1	2	2	10
Total	43	16	16	18	18	111

*0 = no Helicobacter; + = individual organisms, or small groups, covering <1/3 mucosal surface; +++ = large groups of organisms on surface and upper pits of >2/3 mucosal surface; ++ = intermediate density (36, 37)*.

a *Positively stained “cytoplasmic dust” within follicles only, n/a, no enlarged lymph-node seen*.

In the 101 subsequent pigs, chronic inflammation was ubiquitous (mild 34%, moderate 61%, severe 5%) and more severe in presence of HLOs. Active inflammation was associated with eosinophils in 99%, with neutrophils in only 10%. Neutrophil infiltration was associated with cryptitis (all but one case), but not with HLOs. Lymphoid aggregates were found in 41% (low density in 95%, medium in rest). Lymphoid follicles were found in 73%, in medium or high density in two-thirds, and more evident in presence of HLOs. Erosive changes were present in two cases, both without HLOs. Mucosal morphology was confirmed as antral in type in 88 of the 91 subsequent pigs, being body-type in one, mixed body/antral in 2.

16S rRNA gene community profiles indicated that *Helicobacter*-specific sequences were detected in 6 of the 10 caecal-content samples. Metagenomic sequencing of caecal-content from a pig (no. 2), with pangastritis associated with corkscrew organisms and severe antral HLO colonization, confirmed the presence of *H. suis* (raw data: http://www.ebi.ac.uk/ena/data/view/PRJEB25966). Seven contigs representing 0.13% of the estimated *H. suis* genome were obtained following *de novo* assembly. When dataset reads were mapped against an available draft assembly (ADGY01), over 4,000 reads, covering 25% of the genome, mapped.

## Discussion

The much greater relative frequency, *H. suis* to *H. pylori*, in gastric biopsies from PD patients (9), than in biopsies from routine gastroenterology patients, might be an incidental finding of little direct aetio-pathogenic consequence to PD. Excess *H. suis* could be a marker of the immune dysfunction in PD (38). It could also represent expansion of previously hidden infection into a niche left by *H. pylori* eradication (9). Indeed, we report 3 further PD-patients becoming *H. suis*-positive following exposure to *H. pylori* eradication therapy: thus three-quarters of those with *H. suis* had undergone *H. pylori* eradication. Contrary to this, the 12-fold increase in all-cause mortality associated with *H. suis*-positivity suggests it is a malign influence in PD, or a surrogate for one. The sample size is small, yet the magnitude of the effect warrants further investigation, including (as with any observational finding) independent replication. In contrast, *H. pylori*, although associated with increase in deaths from gastric cancer, is not associated with increased all-cause mortality in the general population (17) or (current audit) in PD. However, there is no information on impact of untreated *H. pylori* infection on all-cause mortality in PD.

Our survey of 111 pig stomachs confirms that there is a porcine reservoir for gastric corkscrew organisms in the UK, in line with studies in other countries. Immunostaining on all biopsies, backed-up by 16S rRNA profiling on caecal content from a sample of pigs, confirmed presence of *Helicobacter*. Metagenomic sequencing on caecal content from one pig confirmed presence of *H. suis*.

NHPH infection in humans is well documented (25, 39–42) with animal contact and rural living as risk factors (43–45). Moreover, human NHPH strains have been linked directly, by molecular methods, to infected companion animals (46). Analagously, *H. suis* provides a potential zoonotic candidate to explain the increased PD-specific proportionate mortality ratio in livestock farmers compared with non-farmers (12). In the 6 million decedents studied over a decade (1984–1993) in 26 US states, farmers were predominantly white male (42,857 farming livestock, 191,308 crops), and PD-specific mortality was 19% higher in these white male livestock farmers, 14% lower in crop farmers. In our study, less than half of the *H. suis*-positive PD-patients questioned had direct exposure to pigs or raw porcine products. No study has directly compared PD prevalence according to life-time abstinence, or not, from pork/exposure to pigs. Although the prevalence of PD is low in Arab populations of Israel, Tunisia, Lybia, and Saudi Arabia, it is high in specific rural Israeli Jewish populations (47): potential confounders include ascertainment, smoking, and genetics. Using the converse comparator, those engaged in or retired from an occupational group exposed to pigs and/or raw porcine products, is a practical option. There may, of course, be human-adapted NHPH strains, transmitted (as with most *H. pylori* infections) by close human contact in childhood and persisting. Indeed, the atypical HLO morphology seen here in two patients, who tested positive for both *H. pylori* and *H. suis*, might represent human adaptation.

It is well recognized that NHPH, as well as *H. pylori*, are associated with gastric MALT (a non-Hodgkin) lymphoma, and their eradication with its cure (48, 49). Were *H. suis* causally linked with PD, populations exposed to it might also be at risk of MALT lymphoma. Despite proportionate mortality from malignant neoplasms being less in farmers overall, white male livestock farmers, unlike crop farmers, had greater than expected mortality from non-Hodgkin lymphoma (17%), and acute lymphoid (63%) and myeloid (19%) leukaemias (12). The highest risk of deaths from non-Hodgkin lymphoma or leukemia was in the North Central US, top ranking states in cattle and pig farming, as compared with Northeast, South and West (50). In Canada, incident cases of non-Hodgkin lymphoma in male farm residents (total 1,262 studied) were increased in those exposed to pigs (when ≥13 head), but not to cattle (51), whereas in Finnish farmers incident cases (750) were increased in neither group (52). No information is given about PD and non-Hodgkin lymphoma as comorbidities in the US livestock farmers (12). Lymphoma had not been implicated in any of the deaths reported here.

Clinical and laboratory observations, in line with all-cause mortality, revealed a profile for *H. suis* distinctive from that for *H. pylori. Helicobacter suis* was associated with lower blood pressure, whereas, outside PD, *H. pylori* has been linked weakly (if at all) to hypertension (53). Although orthostatic hypotension is a major problem in PD, we do not suggest that it is directly causal of the excess mortality associated with *H. suis*. However, it might be a biomarker of the causal pathway. Small intestinal bacterial overgrowth, common in PD from presentation (27) was associated here with a lower pulse rate. Low blood pressure and pulse are in keeping with PD dysautonomia (54).

Whilst confirming that *H. pylori* is associated with lymphopenia in PD (38), we associate *H. suis* with a higher circulating T-cell count: there appears to be a differential homeostatic redistribution of T-cells. Compared with *H. pylori* gastritis, NHPH gastritis in man is relatively mild, with less polymorhonuclear and lymphocytic infiltration. *Helicobacter* has been proposed as a trigger for autoimmune gastritis, but gastric atrophy, usually mild to moderate, was found in only 17% of PD patients with biopsy-confirmed *H. pylori* (2). Here, both *Helicobacter* species were associated with the more specific biomarker of autoimmune gastritis, serum intrinsic factor antibody (55). Regarding our third candidate driver, SIBO, we replicate its association with lower blood neutrophil and higher natural-killer cell counts (27). We previously associated lower neutrophils with tremor, higher natural-killer counts with rigidity and brady/hypokinesia. Indeed, there are biological gradients of these measures of PD facets on the respective leucocyte subset counts. We have also described a platykurtic distribution of serum folate in PD (38). Folate concentrations above reference range may be explained by bacterial production in SIBO (56), whilst lower concentrations are linked here with *H. pylori* but not *H. suis*. A lower ascorbic acid in gastric juice and higher pH, consequent on the *H. pylori* gastritis, might impact on dietary folate absorption (57, 58). Platelet volume was higher with *H. pylori*, as described in upper gastrointestinal endoscopy patients (59): whether due to occult blood loss or compensated immune thrombocytopenia is unknown. It was not higher with *H. suis*.

Regarding co-morbidities of PD, *H. pylori* positivity has been weakly linked to cognitive impairment where prevalence is not high (60–62) and, here, the odds for incident cognitive impairment tended to be higher with *H. suis*-positivity. *Helicobacter pylori* eradication has been associated with a decreased risk of progression of dementia (assessed by escalation within a set anti-dementia medication schedule) in a large database study (63) and with lower mortality and improved cognition and function in small open studies (64, 65). Similarly, the severe depression seen here in *H. suis*-positive PD patients might respond better to anti-depressant schedules were *H. suis* eradicated.

In summary, this is the first indication that NHPH infection may be associated with increased all-cause mortality in PD, a disease where peptic ulcer is prodromal (11). With respect to morbidity, it appears that *H. suis* is not entirely a *H. pylori* look-alike, having different immuno-inflammatory and orthostatic cardiovascular outcomes. Differences may reflect *H. suis* having jumped from macaques to find a niche in domesticated pigs, thereby providing a source for zoonosis, whereas *H. pylori* seems as old as anatomically modern humans (66). With better screening tools (such as reliable molecular detection in stool), prospective study of the effect of proven NHPH eradication on facets of PD would become practicable. At present, there is just the one case report on NHPH eradication in PD, but this is encouraging (wheelchair-bound to maintaining independent mobility during 4 years follow-up on stable anti-parkinsonian medication) (7). That PD manifests differently between patients, and the predominance of facets changes within patient over time, suggest an interplay of different drivers and mediators (26). Halting one disease driver may allow another to emerge, as might be the case in the reciprocal relationship between *H. pylori* and *H. suis*. A blanket gut microbiome signature approach, where clusters of co-existing organisms are related to presence or absence of a disease, may be too blunt an instrument to unravel the natural history of this slowly evolving chronic disease. Candidature of NHPH in the PD-specific mortality of livestock (but not arable) farmers (12) provides an alternative to the longstanding, but unsubstantiated, hypothesis on agrochemical usage. Moreover, our study suggests that there is a reservoir of human-adapted *H. suis*, remote in time, space, and person from livestock farming. Zoonosis may prove a vital clue, but the hypothesis generated encompasses human to human transmission.

## Ethics Statement

This is an audit of clinical outcome in patients and as such it does not require ethics committee approval.

## Author Contributions

RD, SD, AC, and CW designed the study and wrote and revised the draft manuscript and subsequent manuscripts. AN, RE, AS, IB, and DT participated in design and coordination of the work and helped to draft and revise the manuscript. AA and RT contributed to reviewing the literature. AA performed the necropsies under supervision of AN. RE was responsible for the molecular microbiology. AS was responsible for the histology with immunostaining in humans and pigs. MI was responsible for the immunology. All authors read and approved the final manuscript.

### Conflict of Interest Statement

The authors declare that the research was conducted in the absence of any commercial or financial relationships that could be construed as a potential conflict of interest.

## Appendix

### Methods

#### Molecular Microbiology (Continued)

Caecal-content DNA was extracted (ExtractMaster Fecal DNA Extraction Kit, Epicentre, Madison, US).

Microbial community composition was assessed by sequencing 16S rRNA gene amplicons. Extracted DNA was amplified with V4 and V5 region universal primers (U515F: 5'-GTGYCAGCMGCCGCGGTA and U927R: 5'-CCCGYCAATTCMTTTRAGT) (32). Fusion primers consisted of the Illumina overhang forward (5'-TCGTCGGCAGCGTCAGATGTGTATAAGAGACAG) and reverse (5'-GTCTCGTGGGCTCGGAGATGTGTAATAAGAGACAG) adapter. Amplification with FastStart HiFi Polymerase (Roche, Diagnostics Ltd, UK) used these cycling conditions: 95 °C for 3 min; 25 cycles of 95 °C for 30 s, 55 °C for 35 s, 72 °C for 1 min; followed by 72 °C for 8 min. Amplicons were purified using 0.8 volumes of Ampure XP magnetic beads (Beckman Coulter). Each sample was then tagged with a unique pair of indices and the sequencing primer, using Nextera XT v2 Index kits, and 2x KAPA HiFi HotStart ReadyMix with these cycling conditions: 95 °C for 3 min; 12 cycles of 95 °C for 30 s, 55 °C for 30 s, 72 °C for 30 s; followed by 72 °C for 5 min. Index-tagged amplicons were purified using 0.8 volumes of magnetic beads. Sample concentrations were measured using the fluorescence-based Quantifluor assay (Promega). Concentrations were normalized before pooling samples, each of which would be subsequently identified by its unique index combination. Sequencing was performed on an Illumina MiSeq with 2 × 300 base-reads according to Illumina's (Cambridge, UK) instructions. Sequence reads were processed by the microbiome-helper pipeline (https://github.com/mlangill/microbiome_helper/). Essentially paired end-reads are merged, based on overlapping ends using PEAR (http://sco.h-its.org/exelixis/web/software/pear/), before data filtering for base-calling quality and amplicon length. Processed sequences are then classified using pick open reference OTUs process implemented in QIIME v1.9.1 (33) against Greengenes 16S rRNA gene database (http://greengenes.secondgenome.com/).

For shotgun metagenome sequencing, an aliquot of DNA extract was fragmented and tagged with sequencing primers and indexes using Nextera XT library preparation kit, sequenced on an Illumina NextSeq with 2 x 150 base-reads. Helicobacter-specific reads were filtered from the dataset using kraken (http://ccb.jhu.edu/software/kraken) and subsequently assembled with Spades.
